# The efficacy and safety of different systemic combination therapies on advanced hepatocellular carcinoma: a systematic review and meta-analysis

**DOI:** 10.3389/fonc.2023.1197782

**Published:** 2023-09-25

**Authors:** Ping Li, Ming Hu, Mei Liu, Xiangyu Ren, Donghong Liu, Jiluo Liu, Jianhua Yin, Xiaojie Tan, Guangwen Cao

**Affiliations:** ^1^ Department of Epidemiology, Second Military Medical University, Shanghai, China; ^2^ Department of Hepatic Surgery, The Third Affiliated Hospital of Second Military Medical University, Shanghai, China

**Keywords:** advanced hepatocellular carcinoma, systemic combination therapy, targeted therapy plus ICI therapy, efficacy, safety

## Abstract

**Background and aims:**

Systemic combinations have recently brought significant therapeutic benefits for advanced hepatocellular carcinoma (aHCC). To design the most effective combination regimens, a systematic review (PROSPERO ID: CRD42022321949) was conducted to evaluate the efficacy and safety of systemic combinations on aHCC.

**Methods:**

We retrieved all the studies from PubMed, Embase, the Cochrane Central Register of Controlled Trials (CENTRAL), and China National Knowledge Infrastructure (CNKI) using the Medical Subject Headings (MeSH) terms until December 21, 2022. The effect indicators (hazard ratio [HR], relative risk [RR], and median) were pooled by a fixed- or random-effects model. A subgroup analysis was conducted according to types and specific therapies.

**Results:**

In total, 88 eligible studies were selected from 7249 potential records. Each kind of combination treatment (chemotherapy plus chemotherapy, targeted plus immune checkpoint inhibitor (ICI) therapy, targeted plus chemotherapy, and targeted plus targeted therapy) had a better objective response rate (ORR) in patients with aHCC, compared to the monotherapy mostly with sorafenib (RR: 1.57 [1.44–1.71]; *I* ^2 =^ 30%). Of those, targeted plus ICI therapy showed better therapeutic efficiency in overall survival (median: 15.02 [12.67–17.38]), progression-free survival (median: 7.08 [6.42–7.74]), and ORR (RR: 1.81 [1.55–2.13]), compared to the monotherapy. Specifically, Atezo plus Beva showed all those benefits. Our pooled result showed all the combinations had increased ≥3 Grade treatment-related adverse events (TrAEs), with an RR of 1.25 [95% CI: 1.15–1.36], compared to the monotherapy.

**Conclusion:**

The systemic combinations, especially targeted plus ICI therapy, including Atezo plus Beva, significantly improve clinical outcomes but increase side effects in patients with aHCC. Future trials should concentrate on improvement in therapeutic efficiency and reduction of toxicity of targeted plus ICI therapy.

**Systematic review registration:**

https://www.crd.york.ac.uk/prospero, identifier CRD42022321949.

## Highlights

• All systemic combinations (chemotherapy plus chemotherapy, targeted therapy plus ICI therapy, targeted therapy plus chemotherapy, and targeted plus targeted therapies) significantly improve the objective response rate in patients with aHCC.
• The targeted therapy plus ICI therapy showed better therapeutic efficiency in overall survival, progression-free survival, and objective response rate, compared to the monotherapy.
• In particular, Atezo plus Beva in targeted therapy plus ICI therapy shows superiority in multiple clinical outcomes over other therapies.
• Increased treatment-related toxicity is evident in combination therapies except for targeted plus chemotherapy.

## 1 Background

Hepatocellular carcinoma (HCC) is one of the most common cancers worldwide, with high incidence and comparable mortality (1). HCC at an early stage can be cured by topical treatments, like resection, liver transplantation, interventional embolization, radiofrequency ablation, and microwave ablation. For moderate-stage HCC, transcatheter arterial chemoembolization (TACE) shows therapeutic efficiency. More recently, some novel embolic materials and technologies have been employed, especially the superstable homogeneous iodinated formulation technology (SHIFT), showing long-term stability and favorable pharmaceutical value (2–5). However, over 50% of patients with HCC are diagnosed at advanced stages and therefore not suitable for surgical or locoregional therapies (6). Patients with advanced HCC, which is regarded as incurable, have limited treatment options and poor prognosis, until the advent of tyrosine kinase inhibitor (TKI) sorafenib for systemic therapy (7). According to the National Comprehensive Cancer Network (NCCN) and American Society of Clinical Oncology (ASCO) guidelines, systemic treatments were recommended for patients with advanced HCC (aHCC) (8, 9). Over the past decades, sorafenib was the leading systemic agent for those patients, followed by lenvatinib as well as other monotherapies (7, 10, 11). Recently, systemic combinations, like immunotherapy and targeted therapy, have brought significant benefits for those patients, which bring great changes to the treatment of advanced HCC (12). For instance, a randomized, phase III trial (EACH) in Asian patients with aHCC, showed progression-free survival (PFS; hazard ratio [HR]: 0.62, 95% confidence interval [CI]: 0.49–0.79), and response rate (8.15% vs. 2.67%, p = 0.02) benefits for FOLFOX4 (infusional fluorouracil, leucovorin, and oxaliplatin) over doxorubicin (13). Another two phase II clinical trials showed sorafenib–oxaliplatin–gemcitabine/capecitabine increased overall survival (OS), objective response rate (ORR), and PFS (14, 15). In a global, phase III trial (IMbrave150), atezolizumab (Atezo) combined with bevacizumab (Beva) resulted in better OS (HR: 0.58 [95% CI: 0.42–0.79]) and PFS (median: 6.8 [95% CI: 5.7–8.3] vs. 4.3 [95% CI: 4.0–5.6] months) than did sorafenib in patients with unresectable HCC (16). More importantly, Atezo plus Beva was listed as a preferred regimen, while sorafenib was listed as another recommended regimen in NCCN guidelines (8). Although systemic combination treatments had great potential to improve the prognosis of aHCC, some phase III trials failed in evaluating systemic combinations for those patients (17, 18). Furthermore, the most critical concern is whether the combination strategy would be a trend in anticancer therapy development and what kind of combinations would be the most optimal one. To facilitate the design of future combination regimens, we performed the systematic review by making an expanded comparison between any two combinations of chemotherapy, targeted therapy, immune checkpoint inhibitor (ICI) therapy, and single certain interventions.

## 2 Methods

### 2.1 Protocol registration

We registered the protocol for this systematic review and meta-analysis on PROSPERO as recommended (https://www.crd.york.ac.uk/PROSPERO, ID: CRD42022321949).

### 2.2 Search strategy

A systematic literature search was performed using PubMed, Embase, the Cochrane Central Register of Controlled Trials (CENTRAL), and China National Knowledge Infrastructure (CNKI) using the Medical Subject Headings (MeSH) terms. The search covered the period from the inception date of each database until December 21, 2022. There was no restriction on publication status or language, and all the included non-English studies were translated into English. The keywords were as follows: (atezolizumab or bevacizumab or sorafenib or oxaliplatin or lenvatinib or pembrolizumab or nivolumab or camrelizumab or apatinib or tyrosine kinase inhibitor* or TKI or PD-1 or PD-L1 or immune checkpoint inhibitor* or ICI) and (hepatocellular cancer or liver cancer or hepatocellular carcinoma or HCC or liver neoplasms*) and (advanced or unresectable or inoperable).

### 2.3 Selection criteria

The inclusion criteria were as follows: 1) studies that evaluated the effects of systemic combination therapies on aHCC, with or without controls; 2) studies that included research subjects with advanced/unresectable HCC; 3) studies that incorporated at least one of available endpoints (overall survival, progression-free survival, or objective response rate). The exclusion criteria were as follows: 1) studies that enrolled patients with cancers that metastasized to the liver, 2) studies with the combination of systemic and topical treatments, and 3) studies that lacked necessary information for data extraction.

### 2.4 Definition of patients, interventions, and endpoints

Patients with aHCC were eligible, defined as advanced metastatic or unresectable hepatocellular carcinoma (i.e., patients with characteristics such as multifocal and/or infiltrative disease within the liver, vascular invasion, or extrahepatic spread), with the diagnosis confirmed by histologic or cytologic analysis or clinical features. Systemic combination therapies are defined as those systemic combination regimens for aHCC, recommended in the NCCN and ASCO guidelines (8, 9), or the actual combinations used clinically, including “atezolizumab+bevacizumab”, “bevacizumab+erlotinib”, “nivolumab+ipilimumab”, “capecitabine+oxaliplatin”, and “sorafenib+GEMOX”. Comparators were the systemic monotherapies including sorafenib, gemcitabine, or oxaliplatin. There was no restriction on the types of control treatment. The primary endpoints were OS at 6 months or longer, PFS at 6 months or longer, and ORR. OS was defined as the interval between the date of random assignment and the date of death from any cause; PFS was defined as the interval between random assignment and progression or death from any cause; ORR was defined as the percentage of patients who had a confirmed complete or partial response. Those endpoints were assessed according to Response Evaluation Criteria in Solid Tumors (RECIST) 1.1. The second endpoints were treatment-related adverse events (TrAEs) and ≥3 Grade TrAEs, according to the National Cancer Institute (NCI) Common Terminology Criteria for Adverse Events (CTCAE) (version 3.0).

### 2.5 Study selection

Our systematic searching was conducted according to the search terms we set before and followed the Preferred Reporting Items for Systematic Reviews and Meta-Analyses guidelines (PRISMA 2020, http://www.prisma-statement.org). The headings and abstracts identified in those databases were reviewed and cross-checked by four investigators (LM, HM, RX, and LD) for the identification of studies that fulfill the eligible criteria. Moreover, reference lists of eligible studies, conference abstracts, and systematic reviews were reviewed to acquire relevant papers as well. For studies using the same data source, the most recent study or the study with the largest sample size was included. Some complicated papers were judged by panel discussion and further arbitrated by the third experienced reviewer (LP).

### 2.6 Data extraction

Prespecified data were independently extracted and double-checked by reviewers (LM, HM, RX, and LD). A standardized data extraction form was designed to manage the necessary items. The following information was extracted from all included publications (papers, abstracts, and reported data of registered trials): title, author, study design, country, sample size, experimental arms, control arms, other demographic characteristics, description of outcomes and adverse events, and their definitions. In particular, a study involving multiple systemic combination therapies was divided into a series of comparisons, each of which contained a group of combination and control regimens.

### 2.7 Statistical analysis

The characteristics of the included studies were synthesized and presented in a tabular form. Categorical variables were presented as counts (%), and continuous variables were described using means or medians. The HR, relative risk (RR), and median with corresponding 95% CI were pooled by the Mantel–Haenszel fixed-effects model if no evidence of significant heterogeneity existed; otherwise, the DerSimonian–Laird random-effects model was applied. The heterogeneity was assessed by *I* ^2^ statistics, classified as low (*I* ^2^ < 25), moderate (25 ≤ *I* ^2^ < 75), and high heterogeneity (*I* ^2^ ≥ 75). Generally, *I* ^2^ statistic >50% was considered significant heterogeneity. The subgroup analysis was conducted by types and specific therapies of systemic combination therapy to detect the effects and heterogeneity of the treatments on aHCC across different combinations. The sensitivity analysis was performed by study design. The publication bias of the included studies was evaluated qualitatively by funnel plot and quantitatively by Egger’s test. These statistical analyses and plots were performed using “meta” and “metamedian” packages from R software, version 3.6.2 (R Foundation for Statistical Computing, Canberra, Austria).

### 2.8 Quality assessment

The Cochrane Collaboration Tools were applied to assess the risk of bias in the included studies. In the tools, the Newcastle–Ottawa Scale (NOS) and version 2 of the Cochrane tool (RoB 2) were used to assess the risk of bias in cohort studies and randomized trials, respectively, as recommended by the Agency for Healthcare Research (19, 20). An Excel tool developed by the agency was applied to implement RoB 2 (https://www.riskofbias.info/welcome/rob-2-0-tool/current-version-of-rob-2). A cohort study was awarded a maximum of nine stars in three sections, and study quality was judged as follows: high risk of bias = 0–3; moderate risk of bias = 4–6; low risk of bias ≥ 7. RoB 2 contains five domains, and each of them was assessed as low risk of bias, some concerns (moderate risk of bias), or a high risk of bias. The judgment principles of the overall bias were as follows: low risk of bias if all domains were labeled as low risk, and high risk of bias if any of the domains were labeled as high risk; otherwise, the assessment result was some concerns. Two reviewers (LM and HM) assessed the risk of bias for each study independently.

## 3 Results

### 3.1 Characteristics of studies and participants with advanced HCC

**Figure 1:**
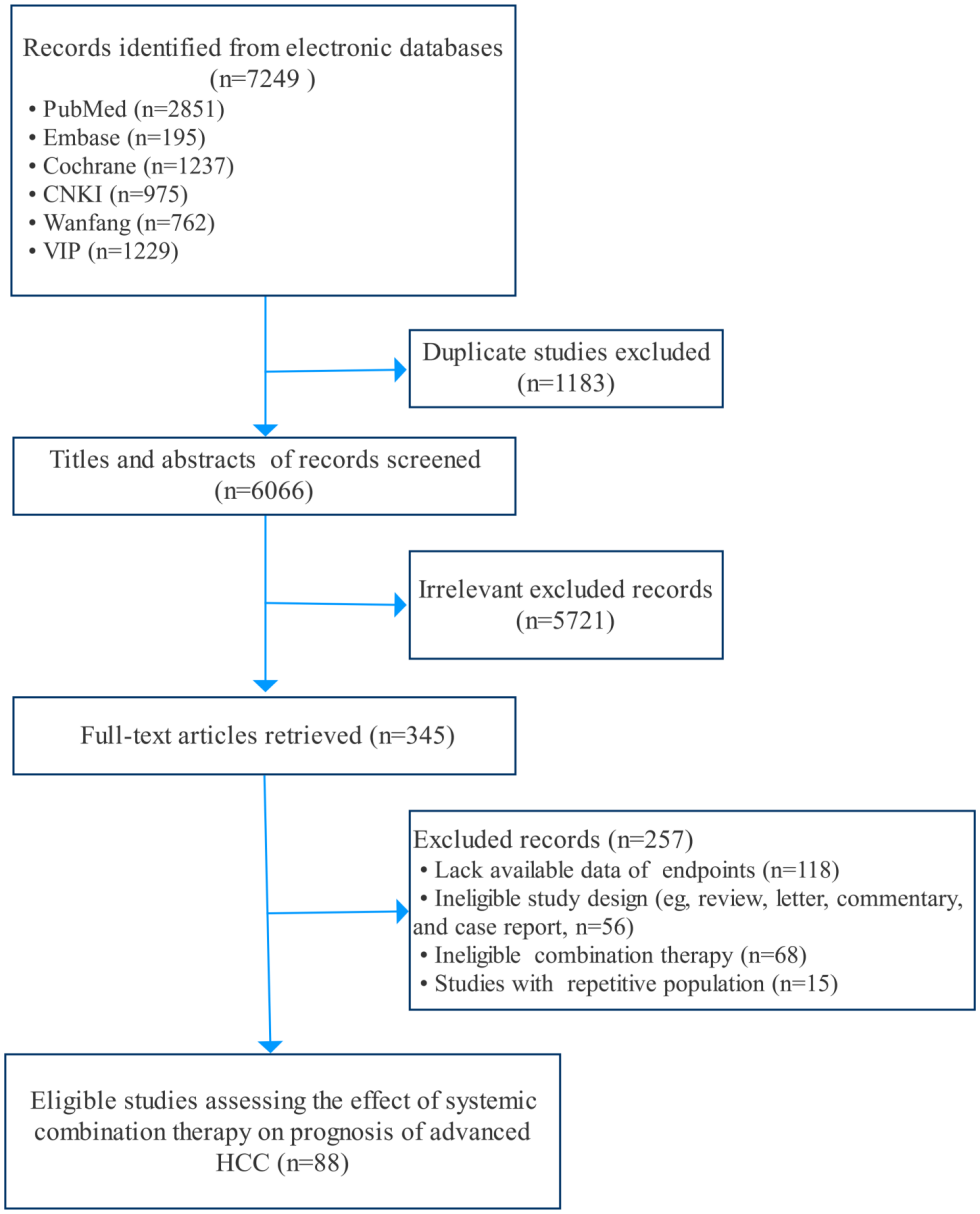
Flowchart of study selection.

In total, 7,249 potential records (papers, abstracts, and registered trials) were screened from databases and other relevant sources. Ultimately, 88 eligible studies (13, 15, 21–107) fulfilling the criteria were included after removing duplicates and reviewing the papers in detail (**Figure 1**). Of those studies, Dhooge 2012 was conducted in both Child-Pugh A and B cohorts; Hitron 2014 and Jiang 2019 contained two kinds of combination regimens, and IMbrave150 included global and China cohorts. Thus, 92 records were incorporated into the analyses. The baseline characteristics of the studies are described and summarized in **Table 1**. In total, 9,748 patients with aHCC from all around the world were included in our systematic review. There were 47 double-arm studies with 7,431 patients and 45 single-arm studies with 2,317 patients. The mean or median age of the total population was 60 years. Most studies were conducted in China (48, 52.2%), and the USA (18, 19.6%). The systemic regimens were the pairwise combinations of targeted therapy, ICI therapy, chemotherapy, and other therapies. Of those, the number and proportions of chemotherapy plus chemotherapy, targeted therapy plus chemotherapy, targeted therapy plus ICI therapy, and targeted plus targeted therapies were 25 (27.2%), 23 (25.0%), 20 (21.7%), and 16 (17.4%), respectively. Subsequently, four specific therapies of those combinations with study numbers greater than 3 were analyzed to probe heterogeneity: Atezo plus Beva, gemcitabine (Gemc) plus oxaliplatin (Oxal), erlotinib (Erlo) plus Beva, and sorafenib (Sora) plus gemcitabine and oxaliplatin (GEMOX). The majority of monotherapies was sorafenib (18, 38.3%), followed by gemcitabine (7, 14.9%). All studies included were trials (73, 79.3%) and cohorts (19, 20.7%).

**Table 1:** The characteristics of studies and participants with advanced hepatocellular carcinoma included in the meta-analysis.

Study	Study design	Country	Experimental arm	Comparator arm	Population size (n)	Age, years (mean or median)	Gender, male (n, %)	Endpoints	Risk of bias
Abou-Alfa 2018 (21)	Trial	USA	Dalantercept+sorafenib	_	21	64	14 (66.7)	OS	High
Abou-Alfa 2019 (22)	Trial	USA	Doxorubicin+sorafenib	Sorafenib	356	62	306 (86.0)	OS and ORR	Low
An 2021 (23)	Trial	China	Tenofovir+sorafenib	Tenofovir	80	54	53 (66.3)	ORR	Moderate
Assenat 2019 (24)	Trial	USA	GEMOX+sorafenib	Sorafenib	48	64	74 (78.7)	OS, PFS, and ORR	Low
Bitzer 2016 (25)	Trial	Germany	Resminostat+sorafenib	Resminostat	45	E: 67; C: 61	38 (84.4)	OS and PFS	High
Cheng 2015 (26)	Trial	USA	CS1008+sorafenib	Sorafenib	109	62	89 (81.7)	OS, PFS, and ORR	Low
Cui 2020 (27)	Trial	China	Oxaliplatin+5-fluorouracil	_	88	67	76 (86.4)	ORR	High
Dhooge 2012 (28)	Cohort	France	Gemcitabine+oxaliplatin	_	17	57	14 (82.4)	OS, PFS, and ORR	Moderate
			Gemcitabine+oxaliplatin	_	15	57	13 (86.7)	OS, PFS, and ORR	Moderate
Du 2019 (29)	Trial	China	Oxaliplatin+epirubicin	Oxaliplatin	120	E: 68; C: 68	65 (54.2)	ORR	Moderate
El 2020 (30)	Trial	USA	Sorafenib+doxorubic	_	30	65	26 (86.7)	OS, PFS, and ORR	High
El-Khoueiry 2018 (31)	Trial	USA	Cixutumumab+sorafenib	_	21	61	17 (81.0)	OS, PFS	High
Feng 2015 (32)	Cohort	Taiwan, China	Sorafenib+cyproheptadine	Sorafenib	52	E: 65; C: 66	45 (86.5)	OS, PFS	Low
Finn 2020 (33)	Trial	USA	Lenvatinib+pembrolizumab	_	100	67	81 (81.0)	OS, PFS, and ORR	High
Gabrielson 2015 (34)	Trial	USA	Temozolomide+veliparib	_	16	62	14 (88.0)	OS and PFS	High
Govindarajan 2013 (35)	Trial	UK	Erlotinib+bevacizumab	_	21	60	13 (61.9)	OS, PFS, and ORR	High
Guo 2017 (36)	Trial	China	Capecitabine+oxaliplatin	Oxaliplatin	54	55	43 (79.6)	ORR	Moderate
Ha 2015 (37)	Trial	USA	Pexa-Vec+sorafenib	Sorafenib	459	61	386 (84.1)	OS and ORR	Low
Han 2020 (38)	Trial	China	Erlotinib+AK105	_	13	58	11 (84.6)	ORR	High
Harding 2020 (39)	Trial	USA	Enzalutamide+sorafenib	Enzalutamide	28	E: 62; C: 70	14 (50.0)	OS, PFS, and ORR	High
He 2018 (40)	Trial	China	Oxaliplatin+5-fluorouracil+leucovorin+sorafenib	_	35	48	28 (88.5)	OS and PFS	High
Hitron 2014 (41)	Trial	USA	BBI608 (napabucasin)+sorafenib	Sorafenib	59	66	76 (78.4)	OS and ORR	Low
			BBI503 (amcasertib)+sorafenib		41	66	76 (78.4)	OS and ORR	Low
Hsu 2010 (42)	Trial	Taiwan, China	Sorafenib+tegafur/uracil	_	53	57	47 (88.7)	OS, PFS, and ORR	High
Hu 2014 (43)	Trial	China	Oxaliplatin+5-fluorouracil	_	22	30-76	19 (86.4)	ORR	High
Huang 2007 (44)	Trial	China	Gemcitabine+oxaliplatin	_	26	51	21 (80.8)	ORR	High
IMbrave150 2021 (45, 46)	Trial	China	Atezolizumab+bevacizumab	Sorafenib	194	56	165 (85.1)	OS and PFS	Low
		Globe	Atezolizumab+bevacizumab	Sorafenib	501	63	414 (82.6)	OS and PFS	Low
Jiang 2019 (47)	Trial	China	Raltitrexed+oxaliplatin	_	27	59	15 (55.6)	ORR	High
	Trial	China	Oxaliplatin+calcium folinate+5-fluorouracil	_	30	57	17 (56.7)	ORR	High
Jin 2013 (48)	Trial	China	GEMOX+interferon α-2a	_	32	54	22 (68.8)	ORR	High
Kim 2020 (49)	Trial	USA	Sorafenib+trametinib	_	17	65	11 (64.7)	OS, PFS, and ORR	High
Li H 2014 (50)	Trial	China	Gemcitabine+oxaliplatin	Gemcitabine	60	50	39 (65.0)	ORR	High
Li J 2016 (51)	Trial	China	Sorafenib+tegafur	Sorafenib	56	63	37 (66.1)	OS, PFS, and ORR	High
Li W 2017 (52)	Trial	China	Gemcitabine+oxaliplatin	Gemcitabine	66	E: 50; C: 50	38 (57.6)	ORR	Moderate
Li Z 2020 (53)	Trial	China	Apatinib+lenalidomide	Apatinib	112	E: 58; C: 59	87 (77.7)	ORR	Moderate
Liao 2015 (54)	Trial	China	Gemcitabine+oxaliplatin	5-fluorouracil	136	E: 67; C: 60	79 (58.1)	ORR	Moderate
Lin 2015 (55)	Trial	China	Sorafenib+GEMOX	Sorafenib	53	51	42 (79.2)	OS, PFS, and ORR	High
Liu 2017 (56)	Trial	China	Gemcitabine+oxaliplatin	Gemcitabine	58	E: 58; C: 58	28 (48.3)	ORR	Moderate
Lu M 2019 (57)	Trial	China	Erlotinib+tegafur	Erlotinib	20	61	17 (85.0)	OS, PFS, and ORR	High
Lu Y 2016 (58)	Trial	China	Gemcitabine+oxaliplatin	5-fluorouracil	65	E: 49; C: 49	43 (66.2)	ORR	Moderate
Niu 2017 (59)	Trial	China	Oxaliplatin+capecitabine	Oxaliplatin	90	_	79 (87.8)	ORR	Moderate
Ogasawara 2014 (60)	Trial	Japan	Capecitabine+peginterferon α-2a	_	24	65	23 (95.8)	OS and ORR	High
Ooka 2014 (61)	Trial	Japan	S-1+sorafenib	_	26	66	23 (88.5)	OS	High
Patt 2017 (62)	Trial	USA	Sorafenib+capecitabine	_	13	65	10 (76.9)	OS	High
Peng 2008 (63)	Trial	China	Gemcitabine+oxaliplatin	Gemcitabine	50	46	31 (62.0)	ORR	Moderate
Petrini 2012 (64)	Trial	Italy	Sorafenib+5-fluorouracil	_	39	67	33 (84.6)	OS, PFS, and ORR	High
Philip 2012 (65)	Trial	USA	Bevacizumab+erlotinib	_	27	60	20 (74.1)	OS, PFS, and ORR	High
Puzanov 2015 (66)	Trial	USA	Tivantinib+sorafenib	Sorafenib	20	62	16 (80.0)	PFS	High
Qin S 2013 (13)	Trial	Asia	FOLFOX4	Doxorubicin	371	E: 50; C: 49	329 (88.7)	OS, PFS, and ORR	Low
Qin S 2019 (67)	Trial	China	Camrelizumab+FOLFOX4/GEMOX	_	34	_	_	PFS and ORR	High
Richly 2009 (68)	Trial	Germany	Sorafenib+doxorubicin	_	18	57	17 (94.4)	ORR	High
Ruanglertboon 2020 (69)	Trial	Australia	Proton pump inhibitors+sorafenib	Sorafenib	542	_	457 (84.3)	OS and PFS	Low
Prete 2010 (70)	Trial	Italy	Sorafenib+octreotide	_	50	68	43 (86.0)	OS and PFS	High
Shahda 2016 (71)	Trial	USA	Lenalidomide+sorafenib	_	5	56	_	OS and PFS	High
Shen E 2013 (72)	Trial	China	5-Fluorouracil+sorafenib	_	39	67	_	OS, PFS, and ORR	High
Sho 2017 (73)	Trial	Japan	5-Fluorouracil+sorafenib	_	12	65	12 (100.0)	ORR	High
Sun 2011 (74)	Trial	USA	Bevacizumab+capecitabine+oxaliplatin	_	40	56	32 (80.0)	OS, PFS, and ORR	High
Tai 2016 (75)	Trial	Singapore	Selumetinib+sorafenib	_	27	63	24 (88.9)	OS and PFS	High
Teng 2021 (76)	Cohort	China	PD-1 inhibitors+lenvatinib	_	24	56	19 (79.2)	OS, PFS, and ORR	Low
Thomas 2018 (77)	Trial	USA	Bevacizumab+erlotinib	Sorafenib	90	61	71 (74.7)	OS	Low
Uchino 2012 (78)	Cohort	Japan	5-Fluorouracil+peginterferon alfa-2a	_	223	64.3	176 (78.9)	OS and ORR	Moderate
Wang F 2014 (79)	Cohort	China	FOLFOX4 or XELOX	_	16	52	14 (87.5)	OS and ORR	Low
Wang Jian 2019 (80)	Trial	China	Gemcitabine+oxaliplatin	Gemcitabine	86	E: 41–70; C: 42–73	41 (47.7)	ORR	High
Wang Jun 2019 (81)	Trial	China	Gemcitabine+oxaliplatin	Gemcitabine	86	E: 50; C: 50	53 (61.6)	ORR	Moderate
Wu X 2021 (82)	Trial	China	Sorafenib+immune checkpoint inhibitors	Sorafenib	54	56	46 (85.2)	PFS and ORR	High
Xu 2019 (83)	Trial	China	SHR-1210+apatinib	_	18	49	17 (94.4)	OS, PFS, and ORR	High
Yang 2015 (84)	Trial	China	Oxaliplatin+gemcitabine	_	30	57	26 (86.7)	PFS and ORR	High
Yau 2012 (85)	Trial	Hong Kong, China	Bevacizumab+erlotinib	_	10	47	7 (70.0)	OS, PFS, and ORR	High
Yau 2013 (15)	Trial	Hong Kong, China	Sorafenib+oxaliplatin+capecitabine	_	51	58	_	OS, PFS, and ORR	High
Yau 2019 (86)	Trial	Globe	Nivolumab+ipilimumab	_	149	_	_	OS, PFS, and ORR	High
Yi 2014 (87)	Cohort	China	Gemcitabine+oxaliplatin	_	36	50	35 (97.2)	PFS and ORR	Low
Yoo 2020 (88)	Cohort	South Korea	Epirubicin+cisplatin+5-fluorouracil	Sorafenib	94	59	70 (74.4)	OS and PFS	Low
Zhang 2018 (89)	Trial	China	Gemcitabine+oxaliplatin	Gemcitabine	58	E: 58; C: 58	28 (48.3)	ORR	Moderate
Zheng 2020 (90)	Trial	China	Apatinib+tegafur	Tegafur	87	E: 56; C: 57	66 (75.9)	OS and ORR	High
Chon 2022 (91)	Cohort	Korea	Atezolizumab+bevacizumab	_	121	63	63 (82.6)	OS, PFS, and ORR	High
D’Alessio 2022 (92)	Cohort	Globe	Atezolizumab+bevacizumab	_	202	69	173 (85)	OS, PFS, and ORR	High
Fulgenzi 2022 (93)	Cohort	Globe	Atezolizumab+bevacizumab	_	296	66	245 (82.7)	OS, PFS, and ORR	High
Hiraoka 2021 (94)	Cohort	Japan	Atezolizumab+bevacizumab	_	171	73	144 (84.2)	ORR	High
Kim 2022 (95)	Cohort	Korea	Atezolizumab+bevacizumab	Lenvatinib	232	E: 62; C: 62	194 (83.6)	OS, PFS, and ORR	Low
Matsumoto 2022 (96)	Cohort	Japan	Atezolizumab+bevacizumab	_	32	77	19 (59.0)	OS, PFS, and ORR	High
Persano 2022 (97)	Cohort	Globe	Atezolizumab+bevacizumab	Lenvatinib	2135	_	1689 (79.1)	OS and ORR	Low
Sasaki 2022 (98)	Trial	Japan	Atezolizumab+bevacizumab	Lenvatinib	68	E: 69; C: 75	53 (77.9)	ORR	Moderate
Fan 2022 (99)	Trial	China	Camrelizumab+lenvatinib	Lenvatinib	126	60	76 (60.3)	ORR	Moderate
Fu 2022 (100)	Trial	China	PD-1 inhibitor+ranvatinib	Ranvatinib	66	61	38 (57.6)	PFS and ORR	Moderate
Gu 2010 (101)	Trial	China	Sorafenib+thymosin α1	Thymosin α1	40	44	35 (87.5)	ORR	High
Wang 2022 (102)	Cohort	China	Sintilimab+lenvatinib	Lenvatinib	75	52	57 (76.0)	OS, PFS, ORR	Low
Wu 2019 (103)	Cohort	China	Sorafenib+arsenic trioxide	Sorafenib	57	56	46 (80.7)	PFS and ORR	Moderate
Yan 2022 (104)	Trial	China	Camrelizumab+Apatinib	Apatinib	68	52	58 (85.3)	OS, PFS, and ORR	Low
Zhao 2021 (105)	Cohort	China	PD-1 antibody+antiangiogenic drug	PD-1 antibody	73	E: 51; C: 57	66 (90.4)	PFS and ORR	Moderate
Zhu 2021 (106)	Trial	China	Sorafenib+camrelizumab	Sorafenib	41	57	28 (68.3)	OS, PFS, and ORR	Moderate
Zong 2017 (107)	Cohort	China	Oxaliplatin+epirubicin	Oxaliplatin	50	E: 60; C: 60	35 (70.0)	OS and ORR	Moderate

E, experimental group; C, control group; OS, overall survival; PFS, progression-free survival; ORR, objective response rate; GEMOX, gemcitabine+oxaliplatin; FOLFOX4, oxaliplatin+calcium folinate+5-Fu; XELOX, oxaliplatin+capecitabine.

### 3.2 Evaluation of efficacy

#### 3.2.1 Overall survival

**Figure 2:**
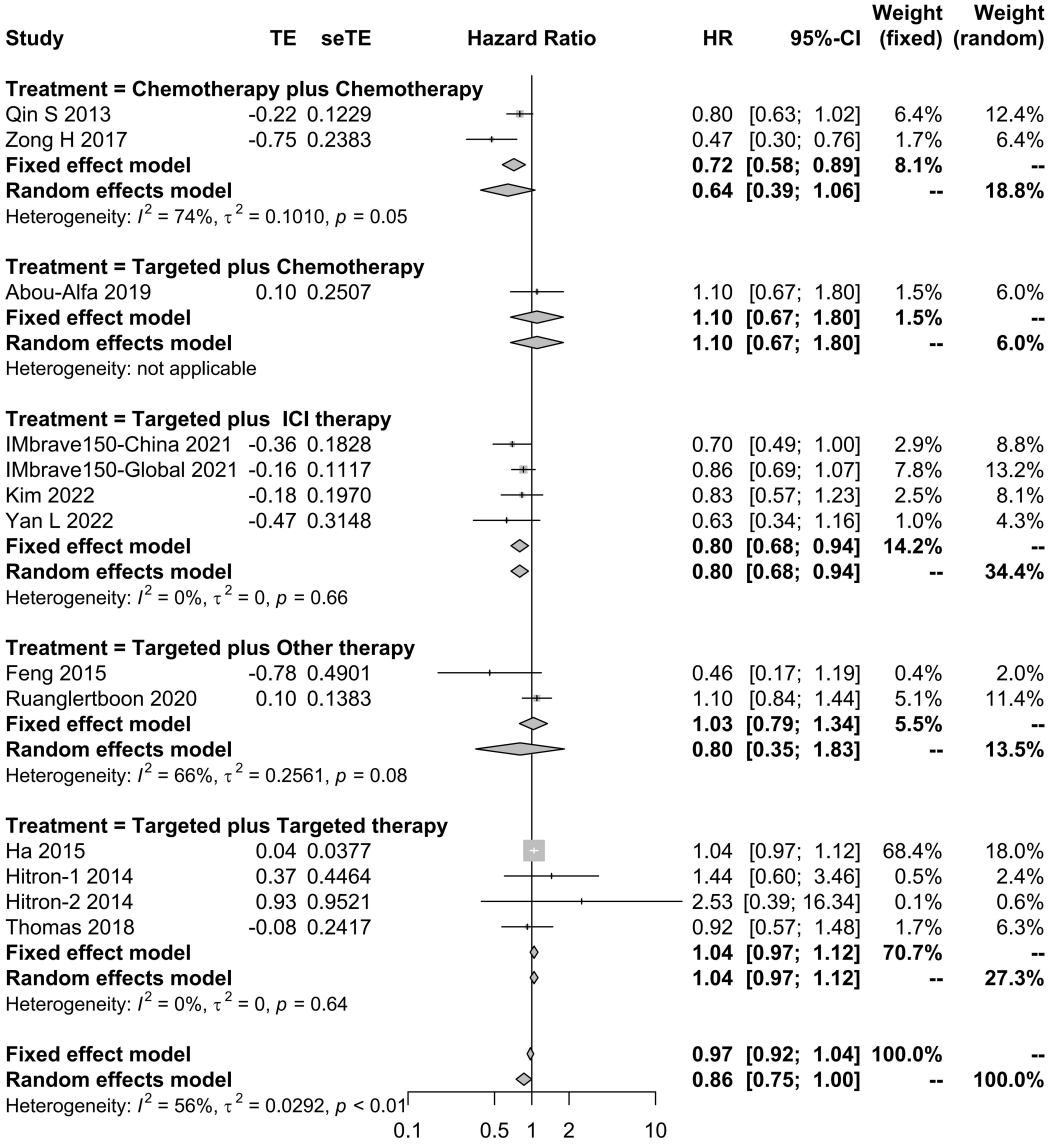
Forest plot for HR of overall survival for the systemic combination therapies, compared to the monotherapy in patients with aHCC. aHCC, advanced hepatocellular carcinoma; HR, hazard ratio; CI, confidence interval.

**Figure 3:**
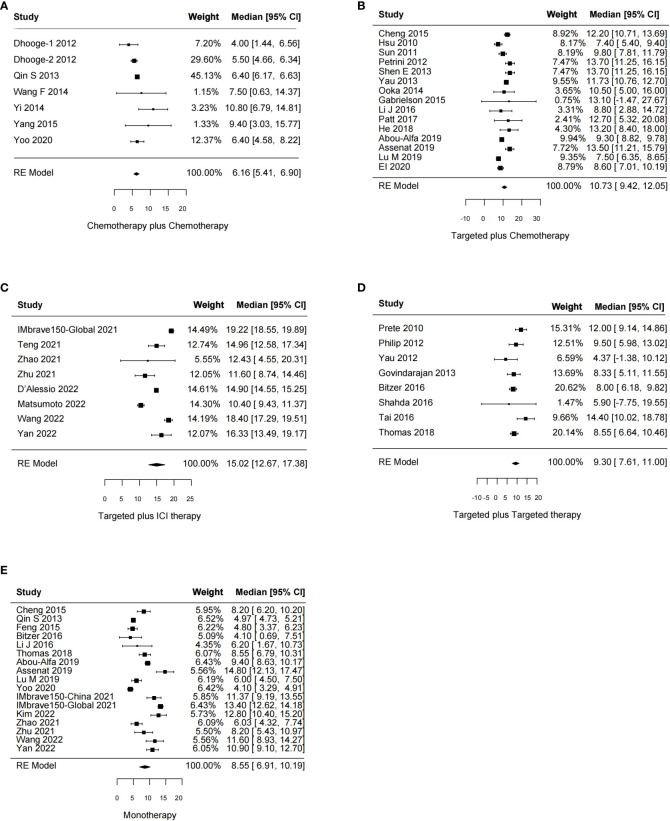
Forest plot for median overall survival of the systemic combination therapies, compared to the monotherapy **(E)** in patients with aHCC. **(A)** Chemotherapy plus chemotherapy. **(B)** Targeted plus chemotherapy. **(C)** Targeted plus ICI therapy. **(D)** Targeted plus targeted therapy. aHCC, advanced hepatocellular carcinoma; CI, confidence interval.

The effects of systemic combination interventions on OS were assessed in a total of 57 studies. In patients with aHCC, targeted therapy plus ICI therapy significantly increased OS (HR: 0.80 [95% CI: 0.68–0.94]; *I* ^2 =^ 0%) and prolonged median OS (15.02 [12.67–17.38] months vs. 8.55 [6.91–10.19] months), compared to the monotherapy. Further, the subgroup analysis of specific therapies indicated this effect was largely due to Atezo plus Beva (HR: 0.81 [0.69–0.96); median: 14.85 [9.87–19.83]). However, the OS benefits were not observed in other types of combinations (**Figures 2**, **3**, **S1, S2**).

#### 3.2.2 Progression-free survival

**Figure 4:**
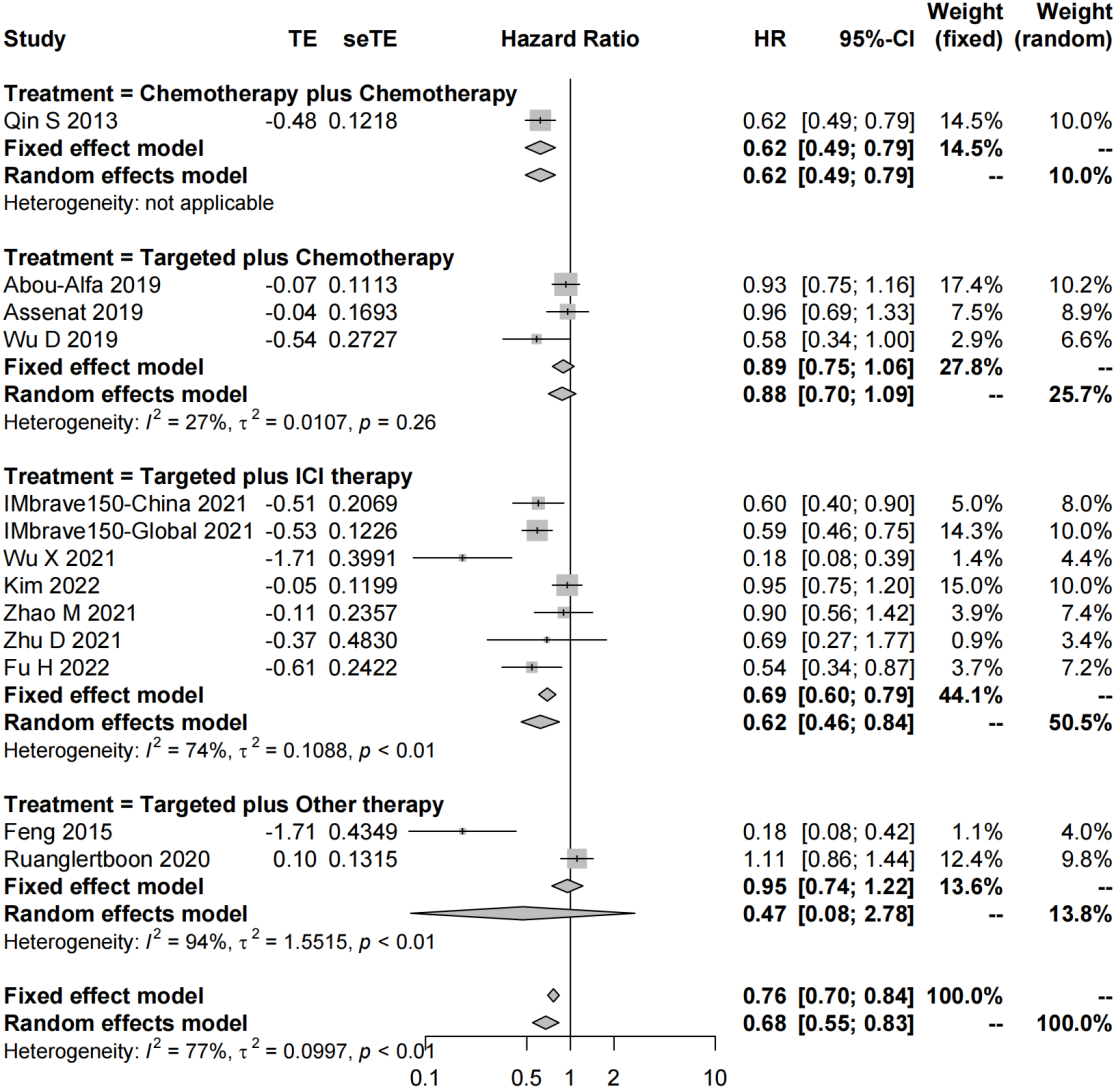
Forest plot for HR of progression-free survival of the systemic combination therapies, compared to the monotherapy in patients with aHCC. aHCC, advanced hepatocellular carcinoma; HR, hazard ratio; CI, confidence interval.

**Figure 5:**
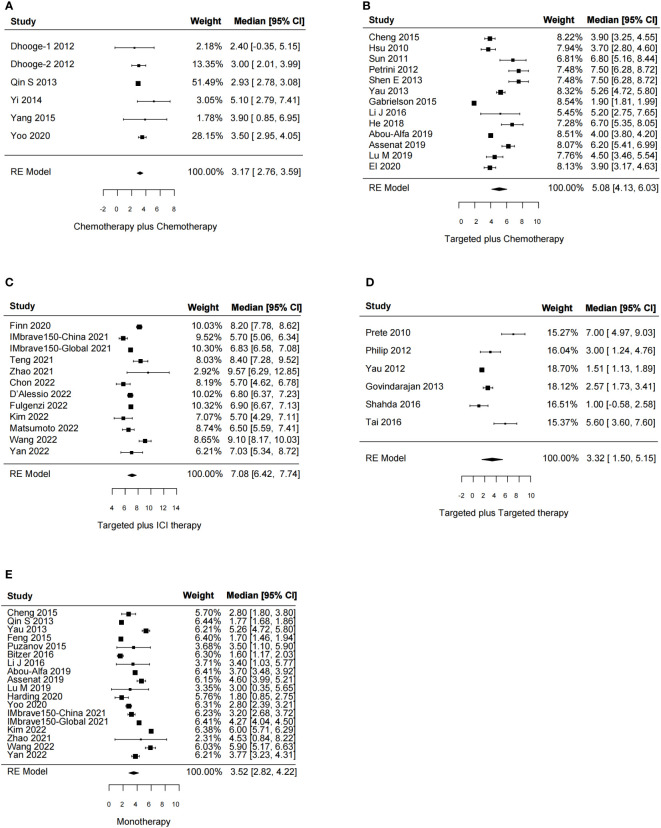
Forest plot for median progression-free survival of the systemic combination therapies, compared to the monotherapy **(E)** in patients with aHCC. **(A)** Chemotherapy plus chemotherapy. **(B)** Targeted plus chemotherapy. **(C)** Targeted plus ICI therapy. **(D)** Targeted plus targeted therapy. aHCC, advanced hepatocellular carcinoma; CI, confidence interval.

In total, 51 studies reported the effect of systemic combination interventions on PFS. The random-effects model indicated that targeted therapy plus ICI therapy had an estimated HR of 0.62 [95% CI: 0.46–0.84], which showed significant PFS benefits over monotherapy. Moreover, the estimated pooled results showed that median PFS was significantly improved if treated with targeted therapy plus chemotherapy (5.08 months [95% CI: 4.13–6.03]) or targeted therapy plus ICI therapy (7.08 months [95% CI: 6.42–7.74]), compared to the monotherapy (3.52 months [95% CI: 2.82–4.22]). Specifically, the median PFS was 5.91 [5.07-6.75] in Sora plus GEMOX and 6.47 [6.06–6.88] in Atezo plus Beva. However, the PFS and median PFS were not improved in the other types of systemic combinations, compared to the monotherapy (**Figures 4**, **5**, **S3, S4**).

#### 3.2.3 Objective response rate

**Figure 6:**
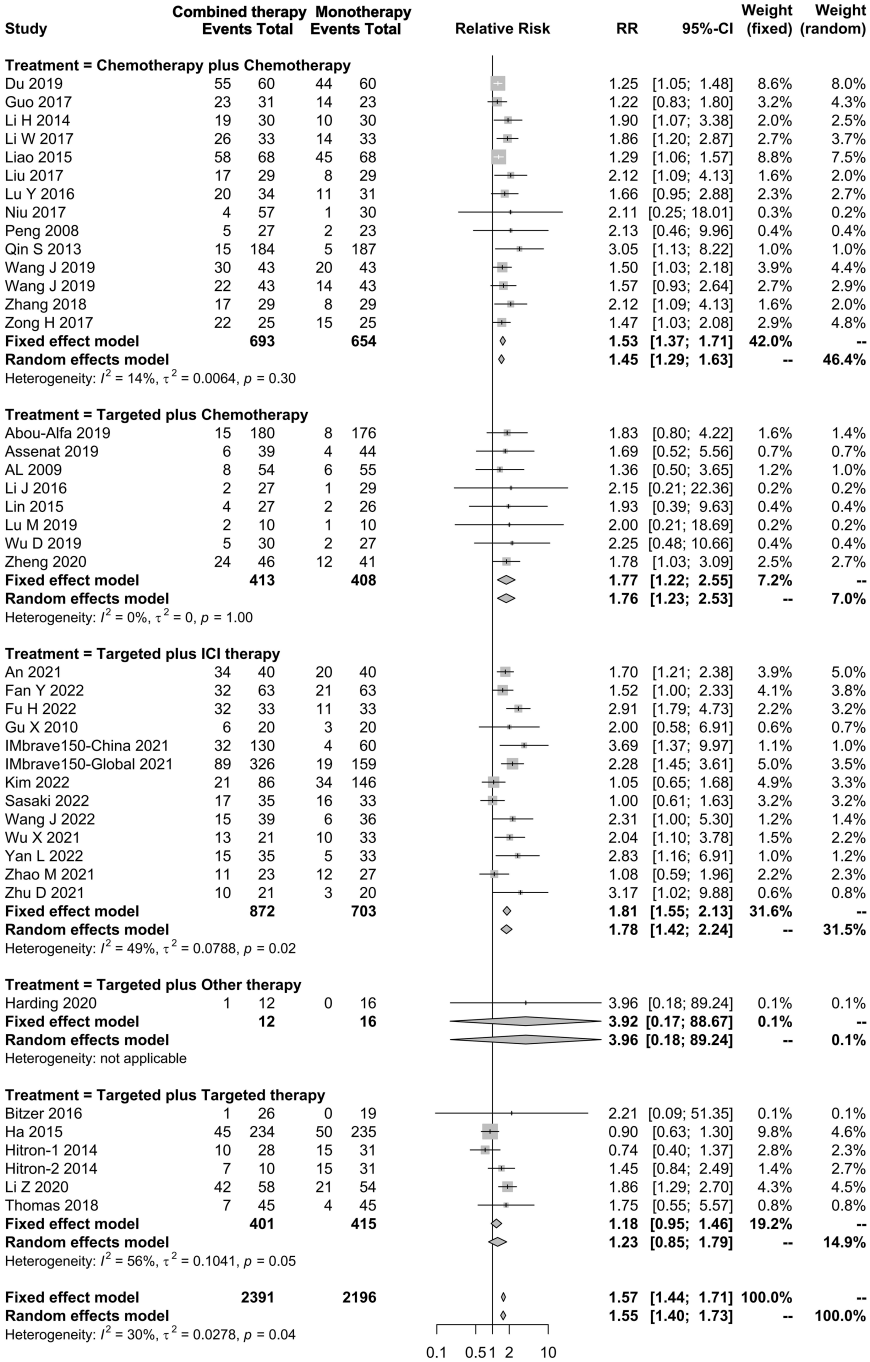
Forest plot for RR of objective response rate of the systemic combination therapies compared to the monotherapy in patients with aHCC. aHCC, advanced hepatocellular carcinoma; RR, relative risk; CI, confidence interval.

In total, 75 studies reported the effect of systemic combination interventions on ORR. Of those, 39 studies included comparisons. The pooled results of combination regimens indicated that the effects across those interventions were consistent, and overall heterogeneity was moderate (RR: 1.57 [95% CI: 1.44–1.71]; *I* ^2 =^ 30%). All systemic combination interventions had an improved ORR in patients with aHCC (chemotherapy plus chemotherapy: 1.53 [1.37–1.71], *I* ^2 =^ 14%; targeted therapy plus chemotherapy: 1.77 [1.22–2.55], *I* ^2 =^ 0%; targeted therapy plus ICI therapy: 1.81 [1.55–2.13], *I* ^2 =^ 49%; targeted plus targeted therapy: 1.23 [0.85–1.79], *I* ^2 =^ 56%), compared to the monotherapy. In the subgroup analysis of specific therapies, Atezo plus Beva, Gemc plus Oxal gained ORR benefits as well (**Figures 6**, **S5**).

### 3.3 Safety assessment

The safety profile of systematic combination therapy was also evaluated in this meta-analysis, including the overall TrAEs in 31 two-arm studies and ≥3 Grade TrAEs in 20 two-arm studies. The incidence rate of TrAEs among those combination interventions was comparable (RR: 1.00 [95% CI: 0.98–1.02]; *I* ^2 =^ 73%; **Figure S6**). For ≥3 Grade TrAEs, the pooled result estimated by the fixed-effects model indicated that the combinations had an increased RR of 1.25 [1.15–1.36], compared to the monotherapy (**Figure S7**). Moderate heterogeneity was detected across those interventions (*I* ^2 =^ 25%). In the subgroup analysis, the incidence rates of ≥3 Grade TrAEs in the chemotherapy plus chemotherapy, targeted therapy plus ICI therapy, and targeted plus targeted were significantly higher, compared to the monotherapy, with RR values of 1.19 [95% CI: 1.01–1.39], 1.26 [0.90–1.75], and 1.38 [1.16–1.64], respectively. However, it was not significant in targeted therapy plus chemotherapy (1.08 [0.93–1.25]).

### 3.4 Quality assessment and publication bias analysis

For quality assessment, the risk of bias in most studies was high or moderate, which should be attributed to nearly half of the studies with a single arm. However, the quality of double-arm studies was generally acceptable, of which the proportion with low or moderate risk of bias was 89.4%. Funnel plots for the effects of systematic combination therapies on OS, PFS, and ORR were asymmetrical. Moreover, the results of Egger’s test indicated that publication bias was detected (OS, p = 0.084; PFS, p = 0.04; ORR, p = 0.002; ≥3 Grade TrAEs, p = 0.092; **Figure S8**).

## 4 Discussion

In this systemic review, we evaluated the efficacy and safety of different systemic combination treatments on the prognosis of aHCC. All kinds of combination treatments (chemotherapy plus chemotherapy, targeted therapy plus ICI therapy, targeted therapy plus chemotherapy, and targeted plus targeted therapies) had better ORRs in patients with aHCC, compared to the monotherapy (**Figure 6**). Importantly, targeted therapy plus ICI therapy, especially Atezo plus Beva, showed superiority in multiple clinical outcomes (OS, PFS, and ORR) over other combinations. Except for targeted therapy plus chemotherapy, all the other combinations had an increased RR for ≥3 Grade TrAEs, compared to the monotherapy. Our findings indicated that the systemic combination regimens had a prominent advantage in treating advanced HCC, although adverse events should be taken into consideration. The pooled results were also calculated separately by study design, and the subgroups with the number of studies greater than 3 were presented. The results of trials and cohorts were generally consistent with studies combined together, indicating the robustness of the pooled results in this study (**Figures S9–S11**). In particular, targeted therapy plus ICI therapy should be given priority on further drug design and development in aHCC.

Previously, several systematic reviews investigated the effects of different systemic treatments on aHCC across lines of therapy (108–110). For instance, a systematic review provided evidence that the combination of PD-1/PD-L1 inhibitors with anti-VEGF agents improved clinical outcomes in patients with aHCC (ORR, p = 0.016; PFS, p < 0.001) but also increased immune-related toxicity (108). The other two network systemic reviews made a comparison between the specific systemic combination therapies and monotherapy (109, 110). It was demonstrated that the Atezo plus Beva combination prolonged OS, PFS, and ORR in patients with unresectable HCC in both the experimental setting and the real world (**Figures S1–S4**). Notably, systemic treatment should be selected based on the goals of individualized treatment. The outcomes of those studies were generally consistent with our findings. However, more clinical trials are needed to update long-term clinical outcomes. Moreover, safety is also an important factor affecting clinical decision-making. Our pooled analysis showed the combinations of chemotherapy plus chemotherapy, targeted therapy plus ICI therapy, and targeted plus targeted therapies had increased and comparable risk of suffering ≥3 Grade TrAEs, which were partly reported in another study (108). The treatment-related toxicity is critical for patients with aHCC.

The mechanisms by which the combination of targeted therapy plus ICI therapy improved the prognosis in aHCC remain largely unknown. Anti-angiogenesis therapy using multikinase inhibitors not only prunes blood vessels essential for cancer progression and metastasis but also has immune modulatory effects by increasing M1 polarization of macrophages and stimulating CD8^+^ T-cell function (111–113). Hence, immune checkpoint blockade and anti-angiogenesis synergistically increase anti-tumor activity in aHCC. However, high dosages of the kinase inhibitors may contribute to immune suppression in the tumor microenvironment (113), indicating that the immune modulatory dosage should be optimized to facilitate the design of future combination regimens. In the precise medicine era, identifying a universal therapy covering a large group is important but not enough. To further improve therapeutic effect, it is of great significance to find out the target patients of those combination treatments. It is reported that investigating treatment-related biomarkers, like immunotherapy, is a promising therapeutic strategy (114–116).

Our study had limitations. First, the heterogeneity existed in total systematic combinations, although types and specific therapies partly accounted for it. Second, some single-arm trials included in this meta-analysis could lead to potential bias. Despite this, the single-arm studies did not cause significant bias in major conclusions since they were only used to estimate the pooled median of OS and PFS. During the process of the study searching, we found an increasing number of studies evaluating the efficacy of new therapy (i.e., Atezo and Beva) in the clinic since 2022. We would exclude single-arm studies when the number of double-arm studies are large enough. Despite those disadvantages, this study provides the most convincing evidence indicating that combinations of systemic therapies especially targeted therapy plus ICI therapy have more advantages compared with monotherapy in treating aHCC.

## 5 Conclusion

Our systematic review and meta-analysis showed that the combinations of chemotherapy plus chemotherapy, targeted therapy plus ICI therapy, targeted therapy plus chemotherapy, and targeted plus targeted therapies significantly improve ORR in patients with aHCC. Furthermore, targeted therapy plus ICI therapy, especially Atezo plus Beva, shows superiority in multiple clinical outcomes over other combinations. Moreover, increased toxicity is evident in combination therapies except for targeted plus chemotherapy. Future trials should concentrate on improvement in the therapeutic efficiency and reduction of the treatment-related toxicity of targeted therapy plus ICI therapy.

## Data availability statement

The datasets presented in this study can be found in online repositories. The names of the repository/repositories and accession number(s) can be found in the article/**Supplementary Material.**

## Author contributions

Conception and design: PL and GC. Administrative support: GC. Provision of study materials or patients: none. Collection and assembly of data: ML, MH, XR, and DL. Data analysis and interpretation: PL and GC. Manuscript writing: all authors. Final approval of manuscript: all authors.
