# Supplementary material for: The efficacy and safety of different systemic combination therapies on advanced hepatocellular carcinoma: a systematic review and meta-analysis
Source: Front Oncol. 2023 Sep 25;13:1197782. doi: 10.3389/fonc.2023.1197782 (PMC10561006; doi:10.3389/fonc.2023.1197782)
Supplement: Supplementary file 1 [file DataSheet_1.pdf]

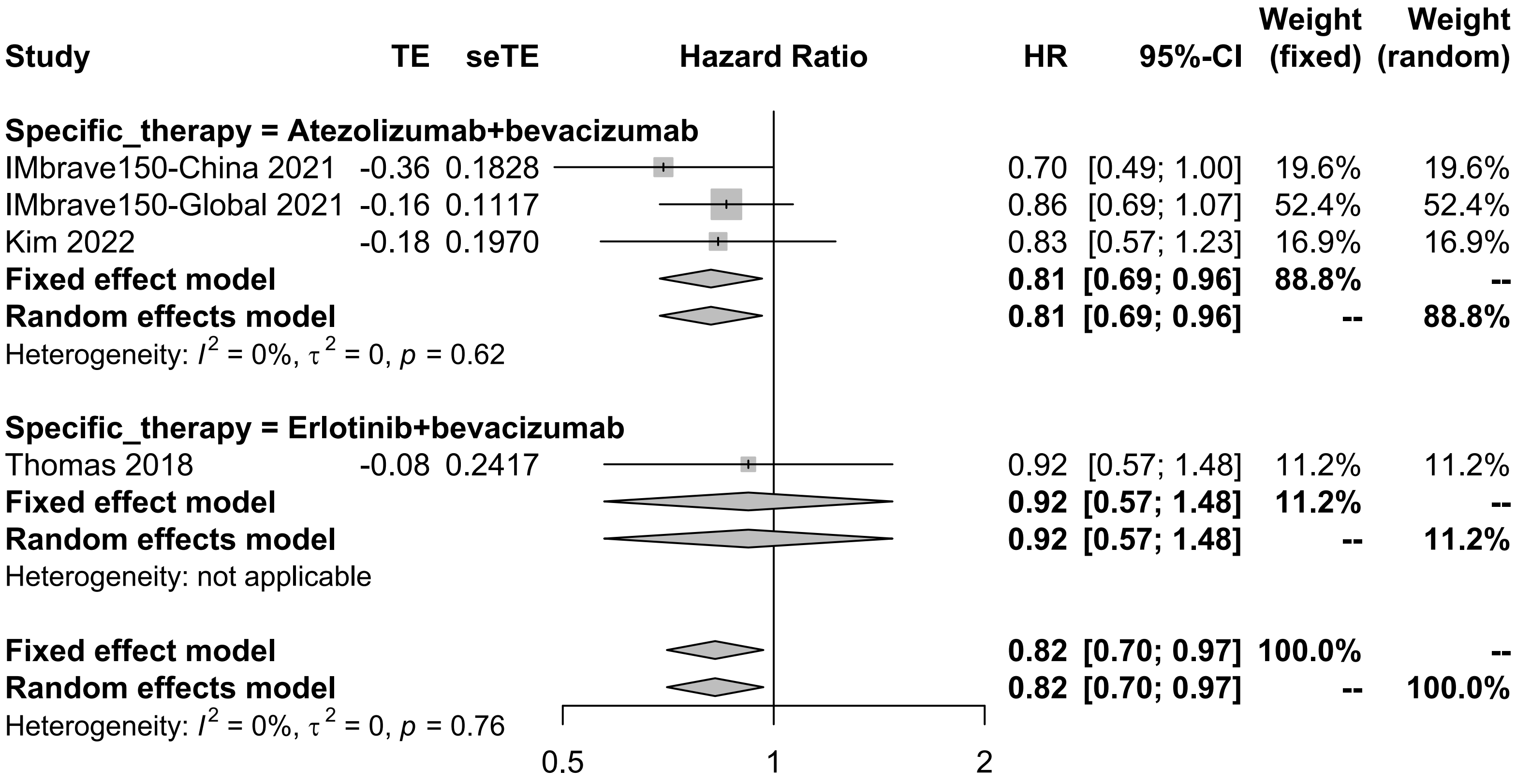

Figure S1. Forest plot for HR of overall survival for the specific therapies, compared to the monotherapy in patients with aHCC

aHCC, advanced hepatocellular carcinoma; HR, hazard ratio; CI, confidence interval.

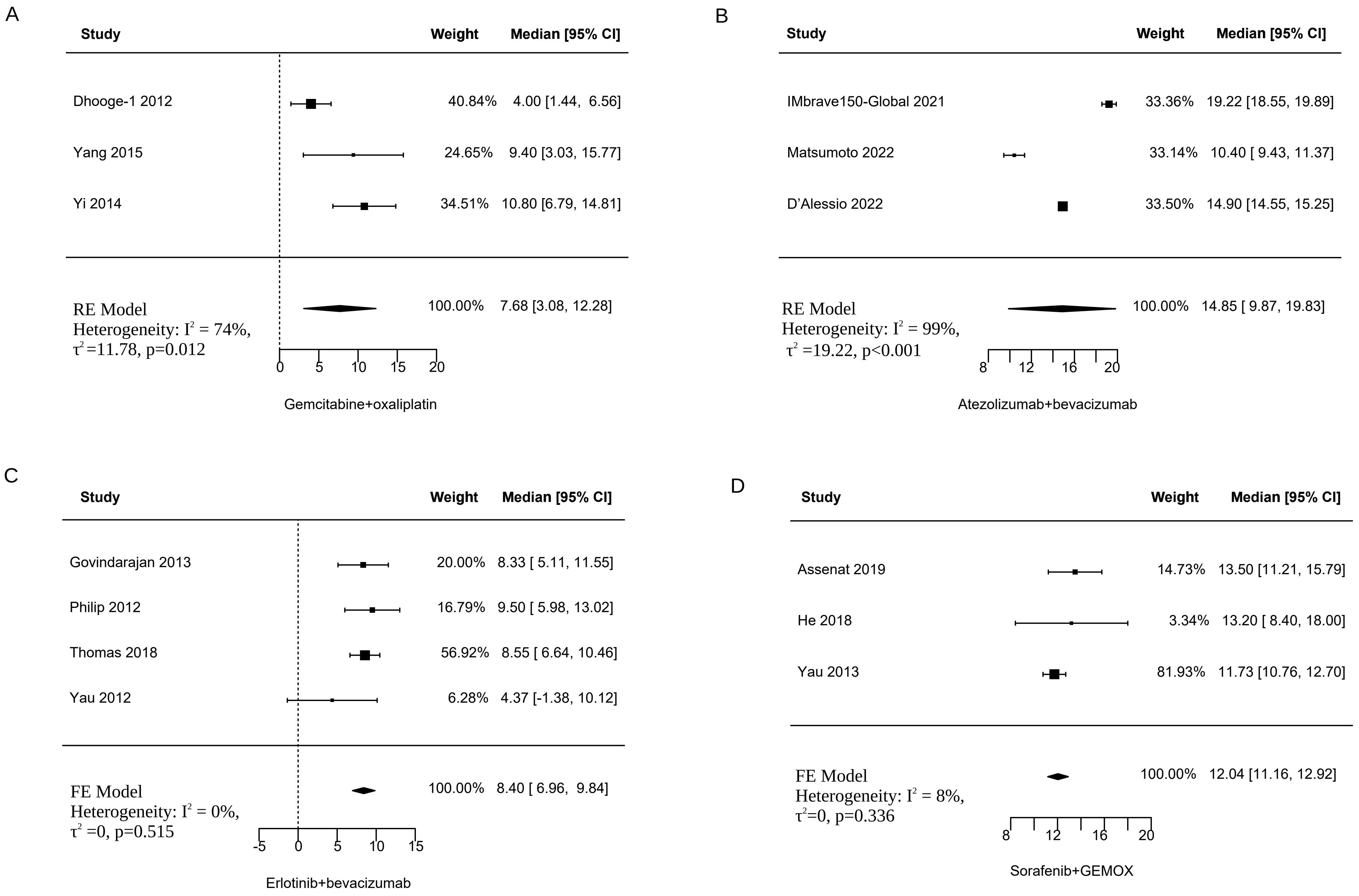

Figure S2. Forest plot for median overall survival for the specific therapies, compared to the monotherapy in patients with aHCC

aHCC, advanced hepatocellular carcinoma; CI, confidence interval.

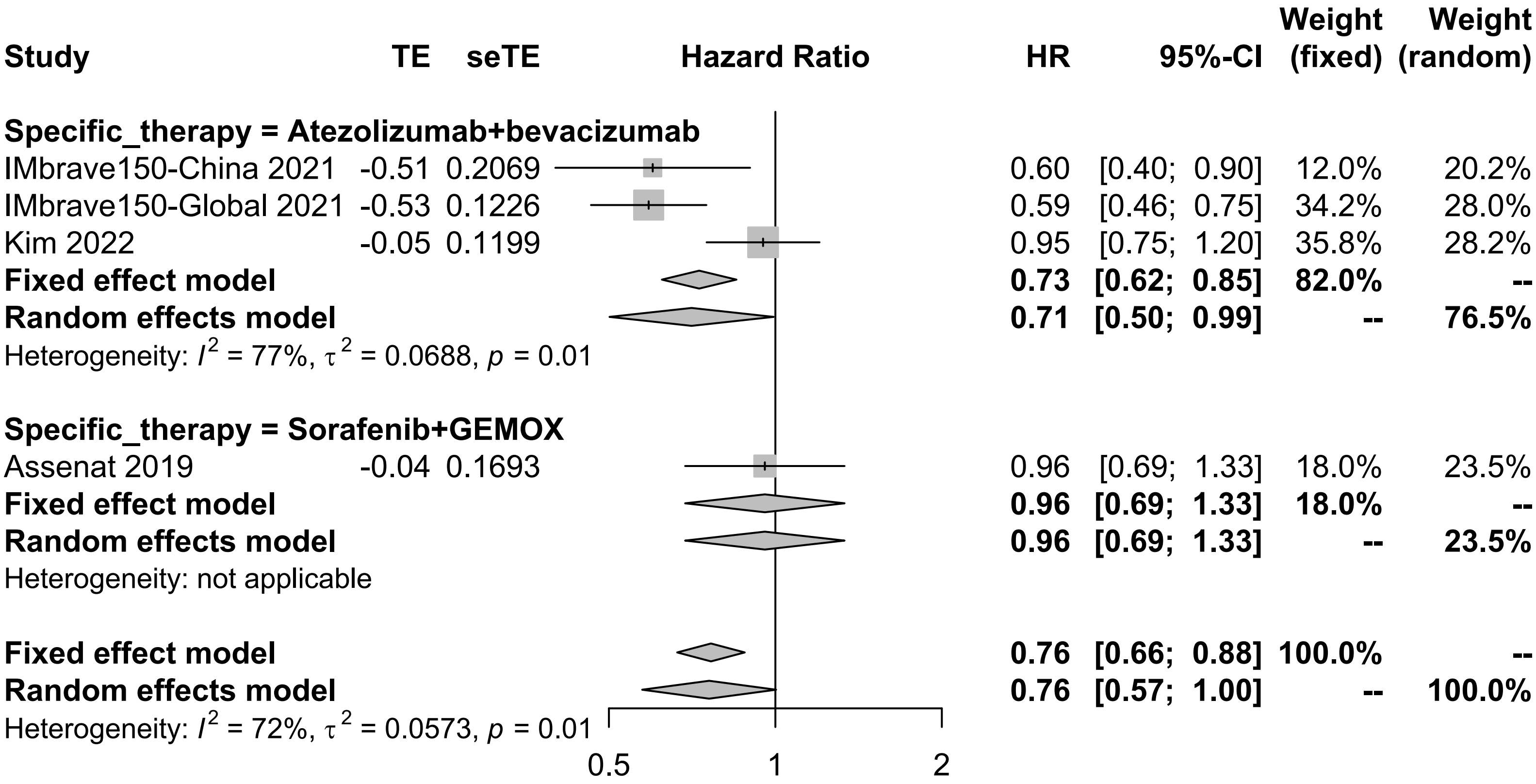

Figure S3. Forest plot of HR for progression-free survival for the specific therapies, compared to the monotherapy in patients with aHCC

aHCC, advanced hepatocellular carcinoma; HR, hazard ratio; CI, confidence interval.

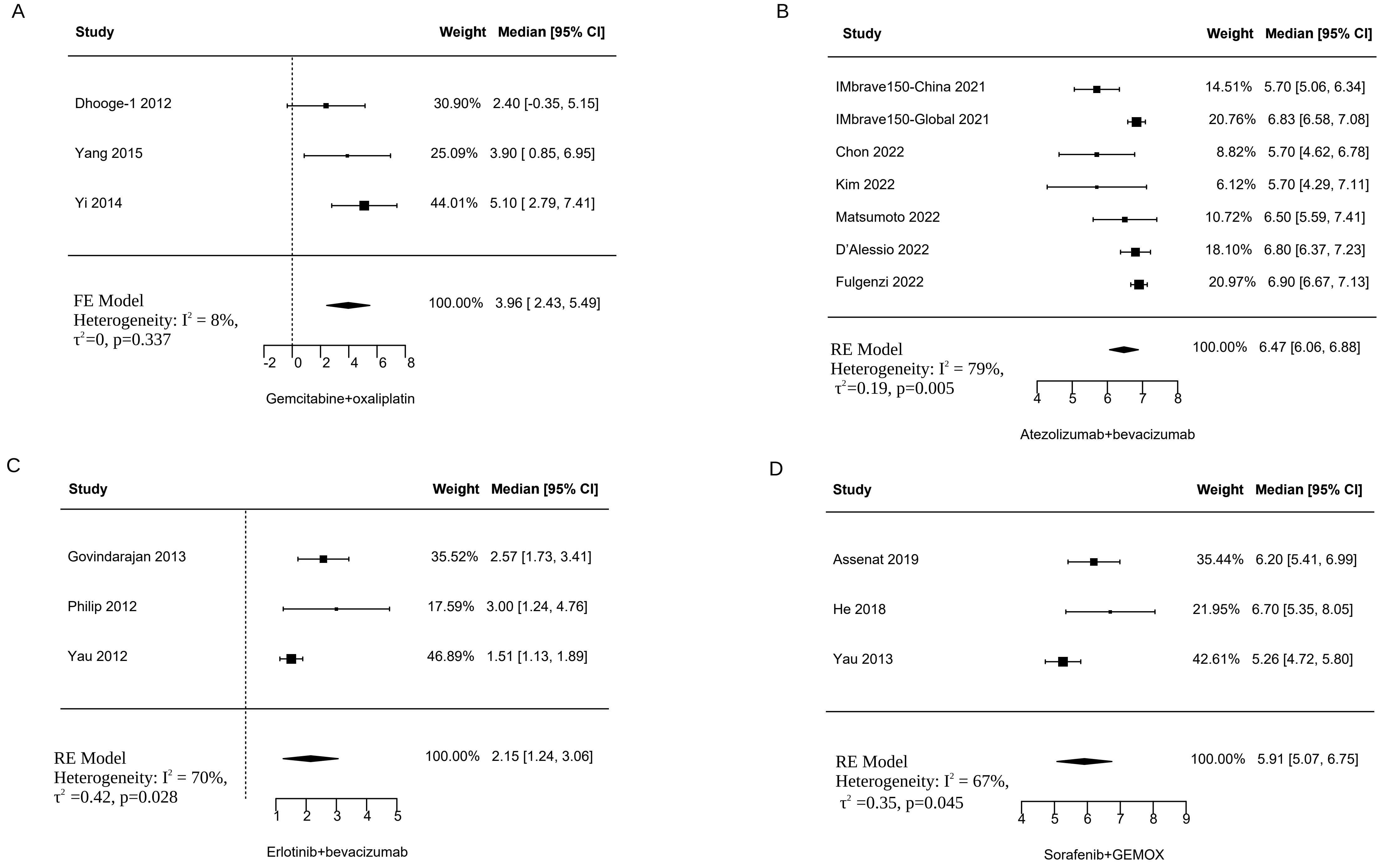

Figure S4. Forest plot for median progression-free survival for the specific therapies, compared to the monotherapy in patients with aHCC

aHCC, advanced hepatocellular carcinoma; CI, confidence interval.

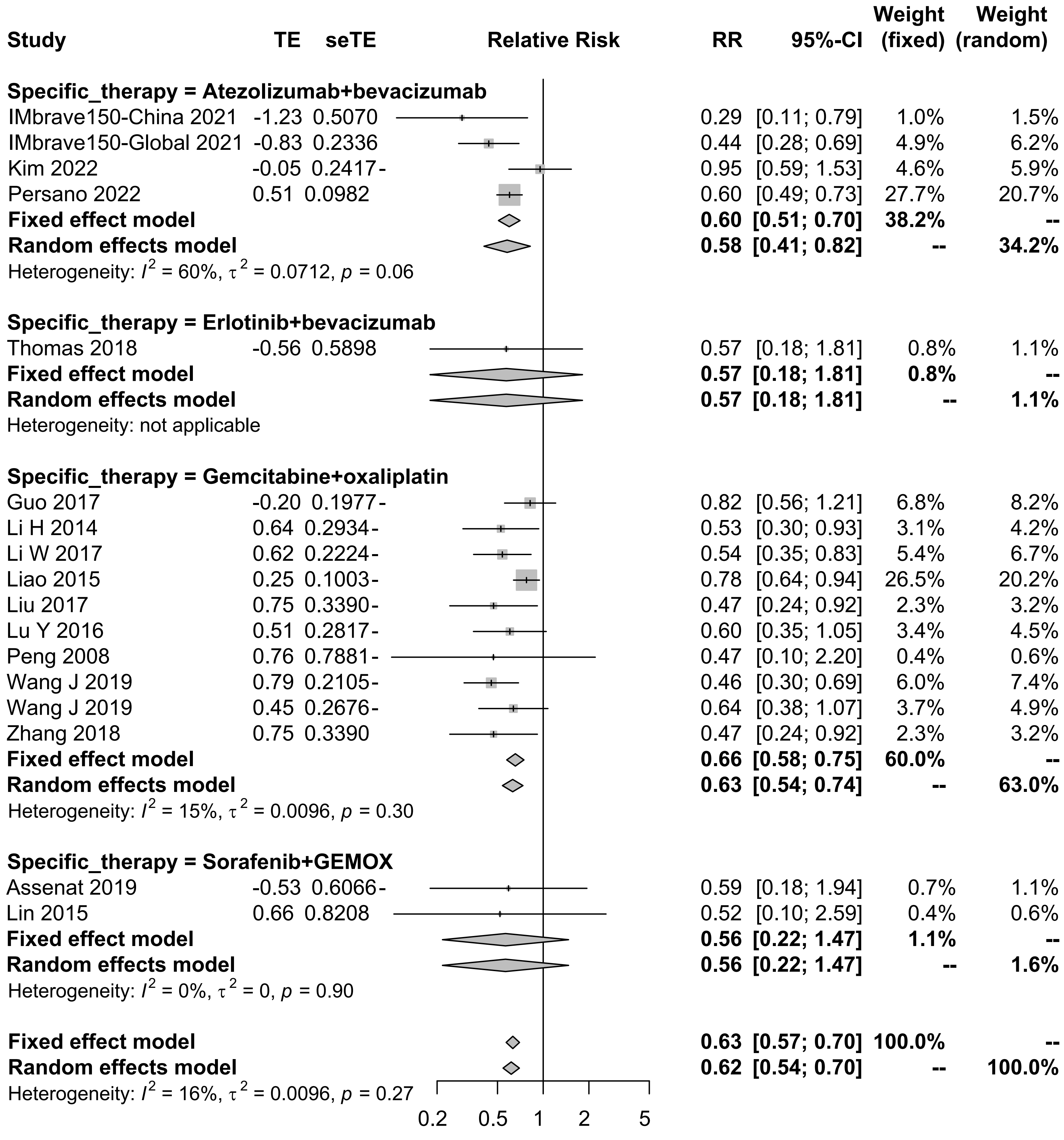

Figure S5. Forest plot for RR of objective response rate of the specific therapies compared to the monotherapy in patients with aHCC

aHCC, advanced hepatocellular carcinoma; RR, relative risk; CI, confidence interval.

Study	Combined therapy Events	Combined therapy Total	Monotherapy Events	Monotherapy Total	RR	95%-CI	Weight (fixed)	Weight (random)
**Treatment = Chemotherapy plus Chemotherapy**								
Du 2019	11	60	21	60	0.52	[0.28; 0.99]	1.3%	0.1%
Guo 2017	31	31	23	23	1.00	[0.93; 1.08]	1.7%	3.5%
Li H 2014	30	30	30	30	1.00	[0.94; 1.07]	1.9%	3.9%
Li W 2017	33	33	33	33	1.00	[0.94; 1.06]	2.1%	4.2%
Liu 2017	29	29	29	29	1.00	[0.94; 1.07]	1.8%	3.8%
Niu 2017	11	60	16	30	0.34	[0.18; 0.65]	1.3%	0.1%
Peng 2008	27	27	23	23	1.00	[0.92; 1.08]	1.6%	3.3%
Qin S 2013	173	183	159	174	1.03	[0.98; 1.10]	10.2%	4.3%
Wang J 2019	2	43	10	43	0.20	[0.05; 0.86]	0.6%	0.0%
Yoo 2020	6	49	15	45	0.37	[0.16; 0.86]	1.0%	0.1%
Zhang 2018	29	29	29	29	1.00	[0.94; 1.07]	1.8%	3.8%
Zong H 2017	4	25	10	25	0.40	[0.14; 1.11]	0.6%	0.0%
**Fixed effect model**		**599**		**544**	**0.90**	**[0.85; 0.95]**	**26.0%**	**--**
**Random effects model**					**0.94**	**[0.86; 1.04]**	**--**	**27.3%**
Heterogeneity: $I^2 = 89\%$, $\tau^2 = 0.0172$, $p < 0.01$								
**Treatment = Targeted plus Chemotherapy**								
Abou-Alfa 2019	167	167	171	171	1.00	[0.99; 1.01]	10.6%	6.6%
Assenat 2019	39	39	44	44	1.00	[0.95; 1.05]	2.6%	4.9%
AL 2009	54	55	54	55	1.00	[0.95; 1.05]	3.4%	4.7%
Lin 2015	27	27	26	26	1.00	[0.93; 1.08]	1.7%	3.5%
Lu M 2019	10	10	10	10	1.00	[0.83; 1.21]	0.7%	0.9%
Zheng 2020	46	46	41	41	1.00	[0.96; 1.05]	2.7%	5.0%
**Fixed effect model**		**344**		**347**	**1.00**	**[0.99; 1.01]**	**21.7%**	**--**
**Random effects model**					**1.00**	**[0.99; 1.01]**	**--**	**25.7%**
Heterogeneity: $I^2 = 0\%$, $\tau^2 = 0$, $p = 1.00$								
**Treatment = Targeted plus ICI therapy**								
Fu H 2022	4	33	15	33	0.27	[0.10; 0.72]	0.9%	0.0%
IMbrave150-China 2021	119	132	54	58	0.97	[0.89; 1.06]	4.7%	2.8%
IMbrave150-Global 2021	310	329	147	156	1.00	[0.95; 1.05]	12.5%	4.9%
Kim 2022	67	86	103	146	1.10	[0.95; 1.29]	4.8%	1.3%
Wang J 2022	39	39	36	36	1.00	[0.95; 1.05]	2.4%	4.6%
Wu X 2021	21	21	33	33	1.00	[0.93; 1.08]	1.6%	3.3%
Yan L 2022	30	35	27	33	1.05	[0.85; 1.29]	1.7%	0.8%
**Fixed effect model**		**675**		**495**	**0.99**	**[0.95; 1.03]**	**28.6%**	**--**
**Random effects model**					**1.00**	**[0.96; 1.04]**	**--**	**17.9%**
Heterogeneity: $I^2 = 37\%$, $\tau^2 = 0.0011$, $p = 0.15$								
**Treatment = Targeted plus Targeted therapy**								
Bitzer 2016	26	26	19	19	1.00	[0.91; 1.09]	1.4%	2.9%
Ha 2015	218	218	214	217	1.01	[1.00; 1.03]	13.5%	6.5%
Hitron-1 2014	20	20	35	36	1.03	[0.97; 1.09]	1.6%	4.5%
Hitron-2 2014	7	7	35	36	1.03	[0.97; 1.09]	0.8%	4.5%
Li Z 2020	58	58	54	54	1.00	[0.97; 1.04]	3.5%	5.6%
Thomas 2018	47	47	43	43	1.00	[0.96; 1.04]	2.8%	5.1%
**Fixed effect model**		**376**		**405**	**1.01**	**[0.99; 1.03]**	**23.6%**	**--**
**Random effects model**					**1.01**	**[1.00; 1.03]**	**--**	**29.1%**
Heterogeneity: $I^2 = 0\%$, $\tau^2 = 0$, $p = 0.88$								
**Fixed effect model**		**1994**		**1791**	**0.97**	**[0.96; 0.99]**	**100.0%**	**--**
**Random effects model**					**1.00**	**[0.98; 1.02]**	**--**	**100.0%**
Heterogeneity: $I^2 = 73\%$, $\tau^2 = 0.0015$, $p < 0.01$								

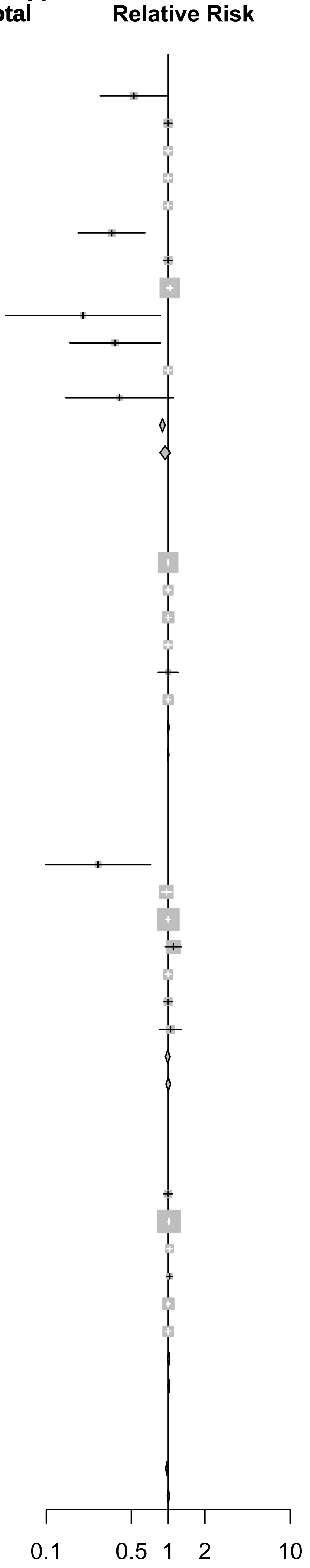

Figure S6. Forest plot for RR of treatment related adverse events of the systemic combination therapies compared to the monotherapy in patients with aHCC

aHCC, advanced hepatocellular carcinoma; RR, relative risk; CI, confidence interval.

Study	Combined therapy Events	Combined therapy Total	Monotherapy Events	Monotherapy Total	RR	95%-CI	Weight (fixed)	Weight (random)
**Treatment = Chemotherapy plus Chemotherapy**								
Guo 2017	9	31	8	23	0.83	[0.38; 1.83]	1.7%	1.5%
Li H 2014	20	30	15	30	1.33	[0.86; 2.07]	2.7%	4.1%
Li W 2017	20	33	21	33	0.95	[0.65; 1.39]	3.8%	5.2%
Peng 2008	18	27	11	23	1.39	[0.84; 2.31]	2.2%	3.3%
Qin S 2013	102	183	79	174	1.23	[1.00; 1.51]	14.7%	11.3%
**Fixed effect model**		**304**		283	**1.19**	**[1.01; 1.39]**	**25.1%**	**--**
**Random effects model**					**1.18**	**[1.01; 1.38]**	**--**	**25.4%**
Heterogeneity: $I^2 = 0\%$, $\tau^2 = 0$, $p = 0.58$								
**Treatment = Targeted plus Chemotherapy**								
Abou-Alfa 2019	68	167	67	171	1.04	[0.80; 1.35]	12.0%	8.7%
Assenat 2019	31	39	32	44	1.09	[0.86; 1.39]	5.5%	9.6%
AL 2009	46	55	43	55	1.07	[0.89; 1.28]	7.8%	12.8%
Lin 2015	7	27	3	26	2.25	[0.65; 7.77]	0.6%	0.6%
Zheng 2020	4	46	4	41	0.89	[0.24; 3.34]	0.8%	0.5%
**Fixed effect model**		**334**		**337**	**1.08**	**[0.93; 1.25]**	**26.6%**	**--**
**Random effects model**					**1.08**	**[0.95; 1.22]**	**--**	**32.3%**
Heterogeneity: $I^2 = 0\%$, $\tau^2 = 0$, $p = 0.82$								
**Treatment = Targeted plus ICI therapy**								
IMbrave150-China 2021	60	132	23	58	1.15	[0.79; 1.66]	5.8%	5.4%
IMbrave150-Global 2021	160	329	51	156	1.49	[1.16; 1.91]	12.6%	9.2%
Kim 2022	37	86	32	146	1.96	[1.33; 2.90]	4.3%	4.9%
Wang J 2022	6	39	9	36	0.62	[0.24; 1.56]	1.7%	1.1%
Yan L 2022	7	35	10	33	0.66	[0.28; 1.53]	1.9%	1.3%
**Fixed effect model**		**621**		**429**	**1.37**	**[1.15; 1.64]**	**26.2%**	**--**
**Random effects model**					**1.26**	**[0.90; 1.75]**	**--**	**21.9%**
Heterogeneity: $I^2 = 61\%$, $\tau^2 = 0.0765$, $p = 0.04$								
**Treatment = Targeted plus Targeted therapy**								
Bitzer 2016	11	26	7	19	1.15	[0.55; 2.41]	1.5%	1.6%
Ha 2015	117	218	77	217	1.51	[1.22; 1.88]	14.0%	10.8%
Hitron-1 2014	10	20	16	36	1.12	[0.64; 1.99]	2.1%	2.6%
Hitron-2 2014	3	7	16	36	0.96	[0.38; 2.44]	0.9%	1.1%
Thomas 2018	25	47	19	43	1.20	[0.78; 1.85]	3.6%	4.3%
**Fixed effect model**		**318**		**351**	**1.38**	**[1.16; 1.64]**	**22.1%**	**--**
**Random effects model**					**1.37**	**[1.15; 1.64]**	**--**	**20.4%**
Heterogeneity: $I^2 = 0\%$, $\tau^2 = 0$, $p = 0.67$								
**Fixed effect model**		**1577**		**1400**	**1.25**	**[1.15; 1.36]**	**100.0%**	**--**
**Random effects model**					**1.21**	**[1.10; 1.33]**	**--**	**100.0%**
Heterogeneity: $I^2 = 25\%$, $\tau^2 = 0.0110$, $p = 0.15$								

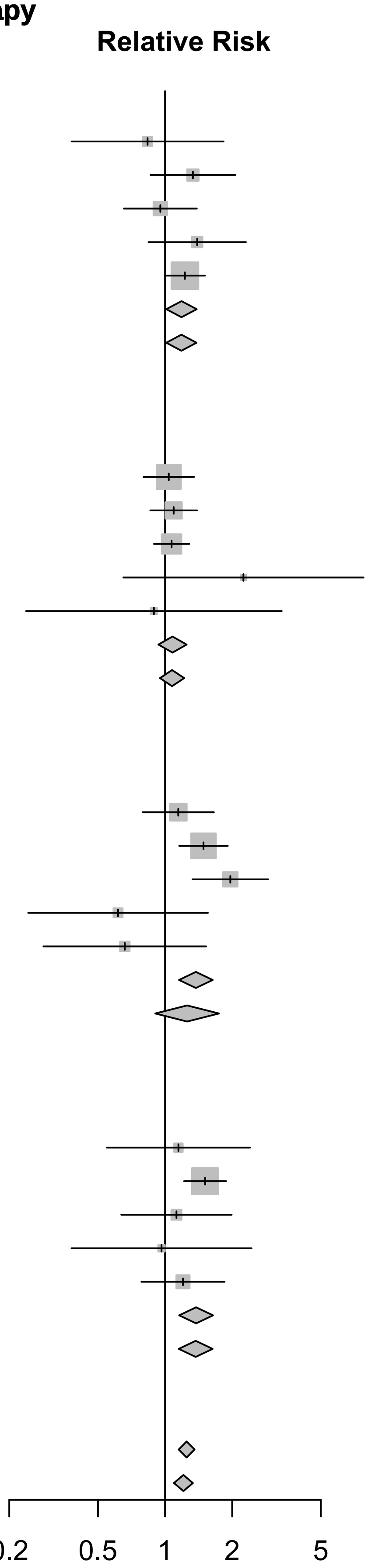

Figure S7. Forest plot for RR of ≥3 grade treatment related adverse events of the systemic combination therapies compared to the monotherapy in patients with aHCC

aHCC, advanced hepatocellular carcinoma; RR, relative risk; CI, confidence interval.

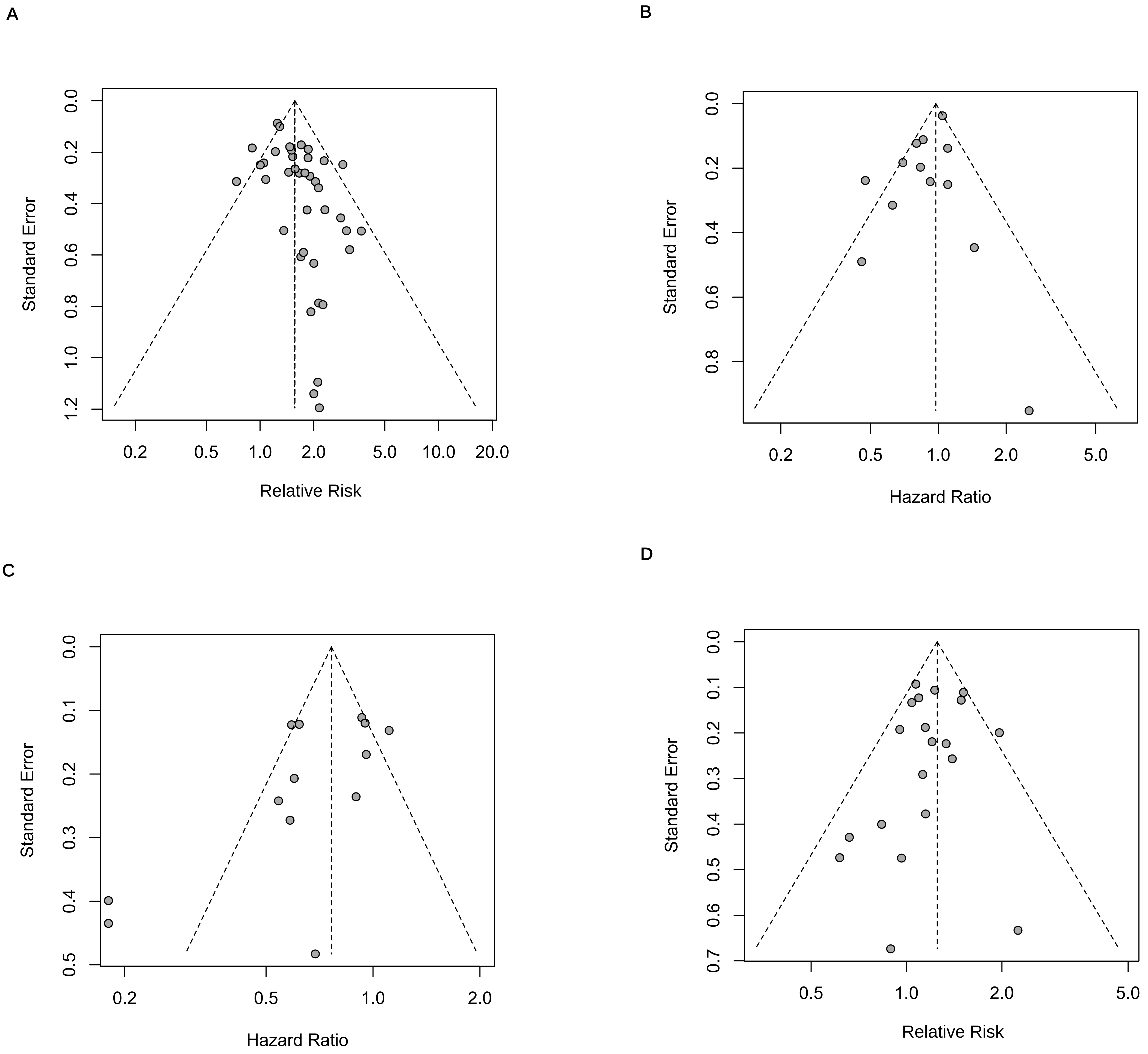

Figure S8. Funnel plot for publication bias. A, funnel plot of ORR; B, funnel plot of OS; C, funnel plot of PFS; D, funnel plot of ≥3 grade treatment related adverse events

ORR, objective response rate; OS, overall survival; PFS, progression-free survival

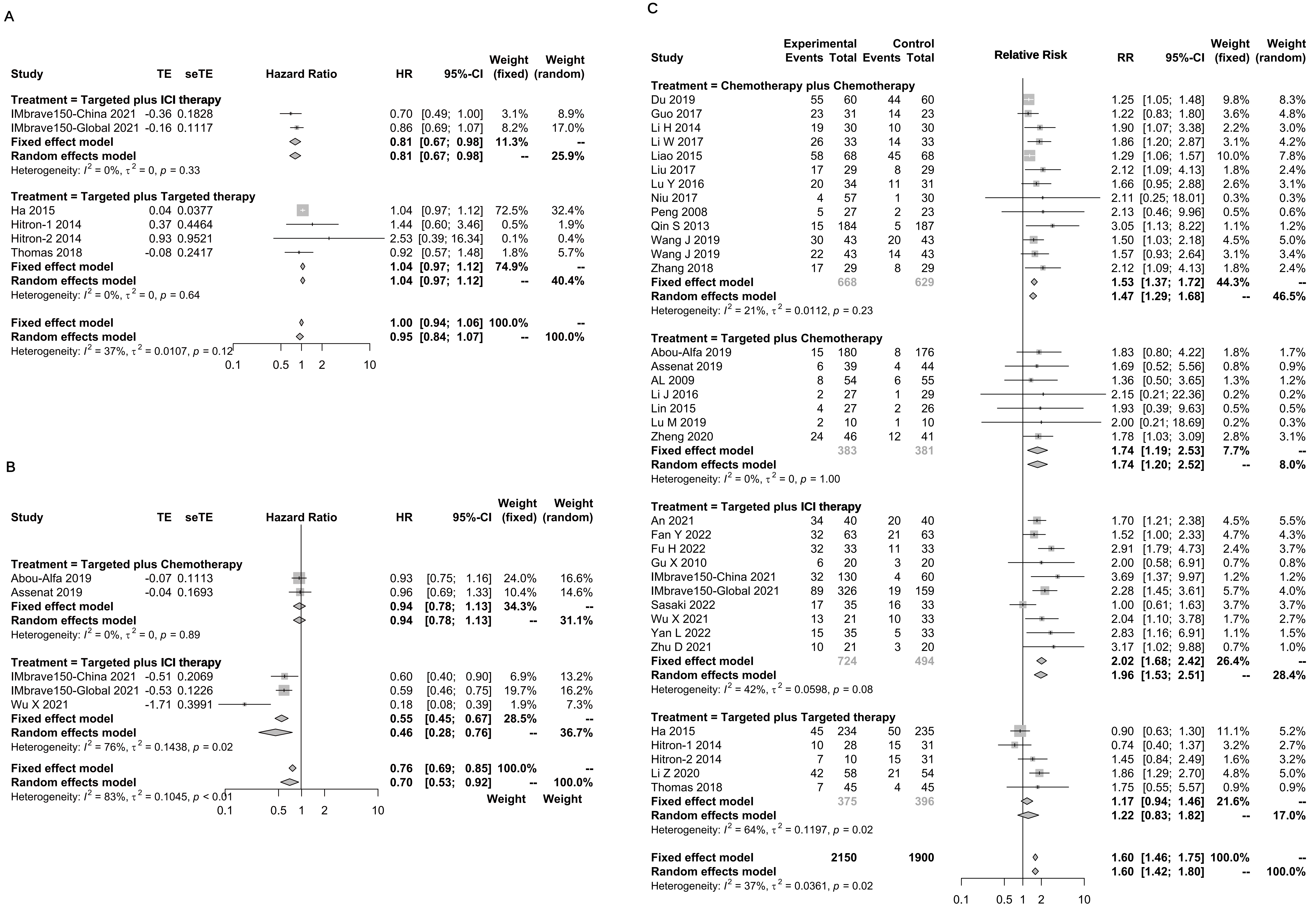

Figure S9. Forest plot in trial studies for overall survival (A) , progression-free survival (B), objective response rate (C) for the systemic combination therapies, compared to the monotherapy in patients with aHCC

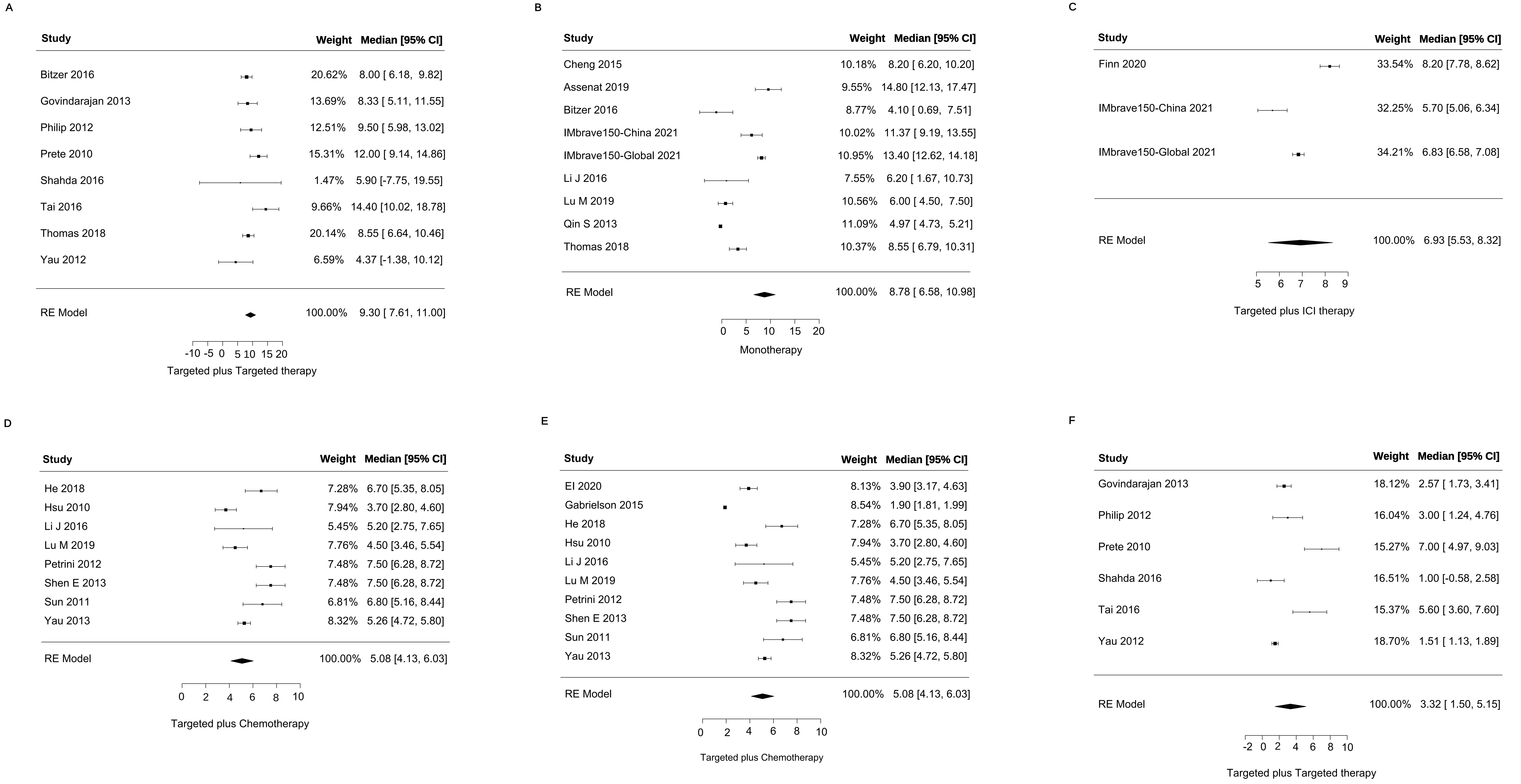

Figure S10. Forest plot in trial studies for median overall survival of targeted plus targeted therapy (A) and monotherapy (B) ; median progression-free survival of targeted plus ICI therapy (C), targeted plus chemotherapy (D) ,targeted plus chemotherapy (E) , and targeted plus targeted therapy (F) in patients with aHCC

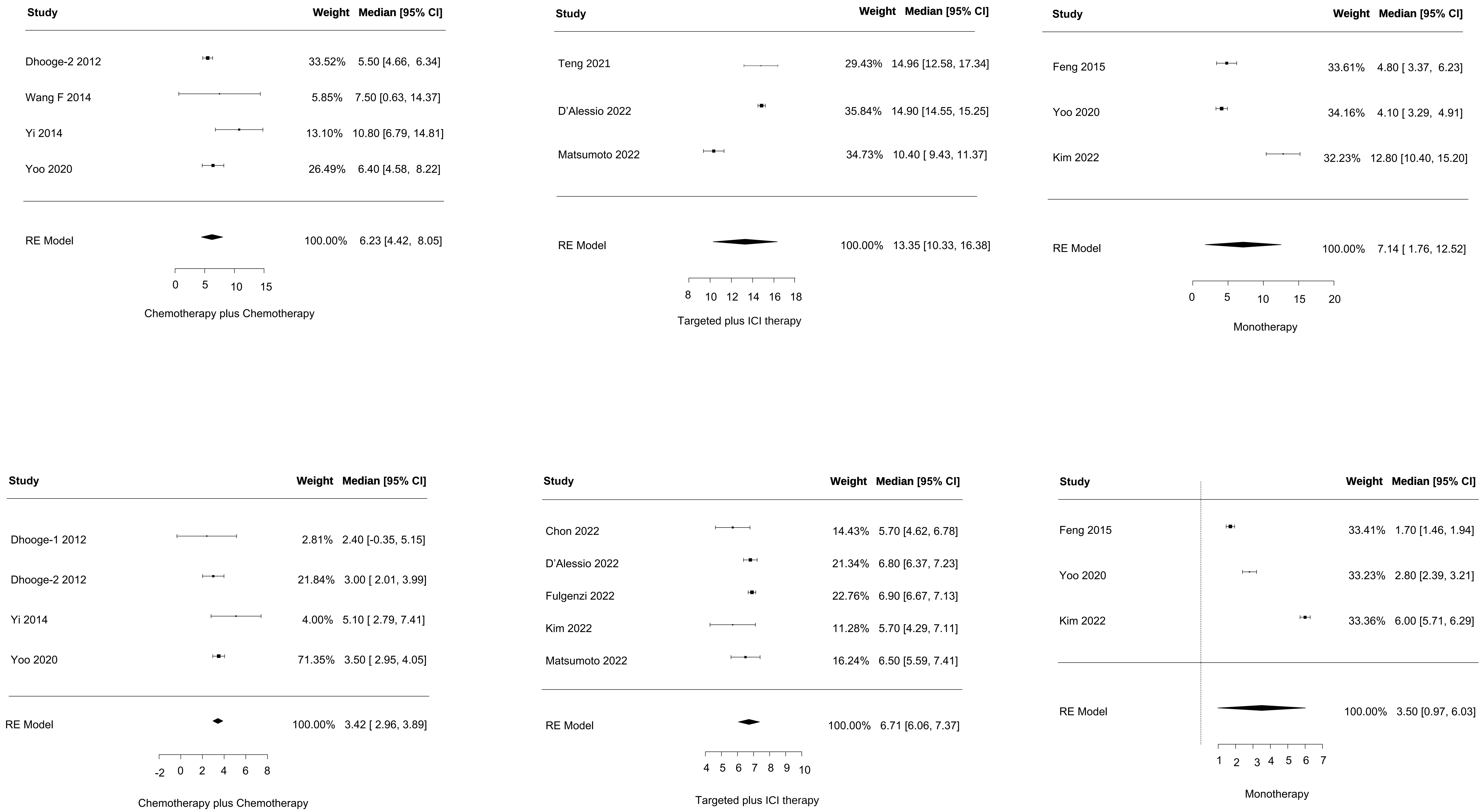

Figure S11. Forest plot in cohort studies for median overall survival of chemotherapy plus chemotherapy (A), targeted plus ICI therapy (B) and monotherapy (C) ; median progression-free survival of chemotherapy plus chemotherapy (D) , targeted plus ICI therapy (E) , and monotherapy (F) in patients with aHCC
